# Identifying gaps in hand hygiene practice to support tailored target audience messaging in Soweto: A cross-sectional community survey

**DOI:** 10.4102/sajid.v37i1.339

**Published:** 2022-03-31

**Authors:** Siobhan L. Johnstone, Nicola A. Page, Michelle J. Groome, Shabir A. Madhi, Portia Mutevedzi, Juno Thomas

**Affiliations:** 1 Centre for Enteric Diseases, National Institute for Communicable Diseases, Johannesburg, South Africa; 2 School of Public Health, Faculty of Health Sciences, University of the Witwatersrand, Johannesburg, South Africa; 3 Department of Medical Virology, Faculty of Health Sciences, University of Pretoria, Pretoria, South Africa; 4 Division of Public Health Surveillance and Response, National Institute for Communicable Diseases, Johannesburg, South Africa; 5 School of Pathology, Faculty of Health Sciences, University of the Witwatersrand, Johannesburg, South Africa; 6 South African Medical Research Council, Vaccines and Infectious Diseases Analytics Research Unit, Faculty of Health Sciences, University of the Witwatersrand, Johannesburg, South Africa

**Keywords:** hand hygiene, risk communication, COVID-19, diarrhoeal diseases, community

## Abstract

Effective risk communication is essential for outbreak mitigation, as recently highlighted during the coronavirus disease 2019 (COVID-19) pandemic. Hand hygiene is one of the proposed public health interventions to protect against severe acute respiratory syndrome coronavirus 2 (SARS-CoV-2) acquisition and transmission along with social distancing, improved ventilation, environmental cleaning, and wearing of masks. Improving hand hygiene practices in the community requires an understanding of the socio-behavioural context. This cross-sectional community survey in Soweto identified gaps in hand hygiene, which can inform appropriate messaging at the community level. Only 42% of survey respondents practiced adequate hand hygiene. Tailored educational messaging should be targeted at young adults in particular, and the importance of soap for hand hygiene must be emphasised for all age groups. Risk communication should expand to focus on preventing multiple infectious diseases during and beyond the COVID-19 pandemic.

## Background

Health communication has played a critical role during the coronavirus disease 2019 (COVID-19) pandemic, highlighting the importance of appropriate risk communication and community engagement (RCCE) in effectively mitigating or controlling outbreaks.^1,2^ Hand hygiene is one component of the bundle of public health interventions^3^ to decrease the risk of severe acute respiratory syndrome coronavirus 2 (SARS-CoV-2) infection and transmission. Furthermore, hand hygiene also prevents or reduces the risk of infection and transmission of many other putative pathogens.^4,5^ Effective hand hygiene is estimated to reduce diarrhoeal episodes by 31% and respiratory illnesses by 21%.^5^ It is estimated that approximately one million diarrhoeal deaths could be prevented globally each year with the universal adoption of adequate hand hygiene involving widely available regular soap products.^6^ Hand sanitisers are shown to be as, if not more effective, at reducing pathogen transmission than regular soap and water.^7^ Although often prohibitively expensive, waterless hand sanitisers are accepted as a feasible alternative in low-income communities with limited soap and water availability, especially during outbreaks.^8,9^

Much remains unknown about how best to encourage good hand hygiene practices at a community level, and specifically how best to sustain these habits in the long term.^4^ Improving hand hygiene practices in settings with good access to sanitation facilities requires changing human behaviour,^10^ which is subject to complexities including existing inequalities, cultural and socioeconomic factors.^3^ Limited understanding of these practices and associated factors in the community is historically responsible for the limited success of promotion campaigns.^10^ Data from South Africa show a significant decline in hand hygiene as a preventive behaviour between the first and second waves of COVID-19 as a result of pandemic fatigue, even amongst people at risk of severe disease.^11^ Creating a culture of good hand hygiene should focus on all levels of society^10^ with RCCE tailored to specific communities based on an understanding of socio-behavioural habits in these communities.^12^ A sustained improvement in hand hygiene practices would have continued benefit during and beyond the COVID-19 pandemic, and health authorities should continue to leverage on the traction gained especially as pandemic fatigue sets in.^11^

We undertook a cross-sectional community survey in Soweto in February 2020, shortly before the first COVID-19 case in South Africa was reported on 05 March 2020.^13^ This report highlights the key findings in terms of hand hygiene practices and factors affecting these practices and identifies gaps for tailored educational messaging to improve hand hygiene practices in this community.

## Methods

### Study area and population

Soweto is an urban township in the south of Johannesburg with an estimated 1.3 million people.^14^ Residents generally have good access to piped water (96.8% have municipal water) and good sanitation (91.6% have flush toilets connected to a sewerage system).^14^ Unemployment is high with 19.0% of households not receiving any set income.^14^

The Soweto Health and Demographic Surveillance Site (HDSS) was established in 2017 through the Child Health and Mortality Prevention Surveillance (CHAMPS) network and currently tracks individuals from 20 778 households in 8 clusters (or sub-places) in Soweto. The methods for this HDSS have been described elsewhere.^15^ This hand hygiene survey was part of a larger survey on diarrhoeal diseases in the community, which used the Soweto HDSS as a sampling frame.^16^

### Sampling methods and data collection

Five hundred households were randomly selected from four of the Soweto HDSS clusters (clusters selected using probability proportional to size sampling). Fieldworkers visited selected households and explained the study to an adult (≥ 18 years of age) representative of the household. Written informed consent was obtained and respondents were interviewed by fieldworkers, in the preferred language of the respondent.

Respondents were asked about water sources in the household, how they usually wash their hands (use of soap and water) and how often they wash their hands at the following critical times: after using the toilet, before preparing food, before eating, before feeding young children and after changing children’s nappies or diapers (where applicable). Households were considered a non-response if they were not available after two visits on two separate days.

### Statistical analysis

Demographic and socioeconomic information for consenting households was matched to the Soweto HDSS database before being de-identified. The International Wealth Index (IWI)^17^ was used as a composite measure of material wealth, combining household assets, housing floor material, toilet facility, number of rooms, electricity access and water source, for each household. An aggregate variable describing hand hygiene as adequate or inadequate was generated from the responses to the questions on hand hygiene. A respondent was described as having adequate hand hygiene if they reported washing with soap and water (as opposed to water only) at all critical times (after using the toilet, before preparing food, before eating and for households with small children; before feeding young children and after changing children’s nappies or diapers). Data were specified as three-level, complex survey data to account for the cluster design of the study. Logistic regression was used to determine risk factors for inadequate hand hygiene. Stata^®^ software (version 14) was used for data analysis.

### Ethical considerations

This study was approved by the Human Research Ethics Committee (Medical) of the University of the Witwatersrand (approval number: M190663) and the CHAMPS Soweto HDSS Community Advisory Board. Written informed consent was obtained from all included participants. All methods were carried out in accordance with relevant guidelines and regulations.

## Results

A total of 374 households (including 1640 individuals) were enrolled. All respondents reported having a facility to wash their hands on the property, with 52% (*n* = 193) having a tap or basin in the room housing the toilet and 48% (*n* = 181) having a tap that was not in the room housing the toilet (either outside the dwelling or in the kitchen). The majority of respondents reported that they always washed their hands after going to the toilet (*n* = 331, 88.5%), before preparing food (*n* = 299, 80.6%), before eating (*n* = 293, 78.6%), before feeding babies and young children (71/92, 77.2%) and after changing children’s nappies or diapers (62/78, 79.5%). Only 64.2% (*n* = 240) reported using soap and water to wash their hands whilst the rest (*n* = 134, 35.8%) used water only. Although specific questions on the use of waterless hand sanitiser were not included, respondents were asked if they use anything other than soap and water to wash their hands. No respondents reported using waterless hand sanitiser. The aggregate variable for adequate hand hygiene indicated that only 42% (*n* = 158) of respondents practiced adequate hand hygiene.

Gender, having children in the household, living in an informal dwelling and IWI did not affect hand hygiene practices. The odds of inadequate hand hygiene decreased significantly with increasing age of respondent (odds ratio [OR] = 0.49, 95% confidence interval [CI]: 0.23–1.02; OR = 0.37, 95% CI: 0.17–0.81; OR = 0.60, 95% CI: 0.29–1.25; OR = 0.34, 95% CI: 0.18–0.63; for age groups 30–39, 40–49, 50–59, ≥ 60 years compared with < 30 years, respectively). The odds of at least one diarrhoeal episode in the past two weeks were higher for households reporting inadequate hand hygiene (OR = 1.86, 95% CI: 1.09–3.17) and tended to be higher for households with a basin in a different room to the toilet (OR = 1.47, 95% CI: 0.97–2.23) (Table 1).

**TABLE 1:** Risk factors associated with inadequate hand hygiene.

Characteristics	*N*	*n*	%	OR	95% CI	*p*
**Gender**						
Male	122	76	62.3	Referent	Referent	-
Female	252	140	55.6	0.76	0.49–1.18	0.217
**Children < 5 years old in the house**						
No	247	139	56.3	Referent	Referent	-
Yes	127	77	60.6	1.20	0.77–1.85	0.420
**Children between 5-15 years old in the house**						
No	200	112	56.0	Referent	Referent	-
Yes	174	104	59.8	1.17	0.77–1.76	0.462
**Dwelling**						
Formal	290	165	56.9	Referent	Referent	-
Informal	65	42	64.6	1.38	0.79–2.42	0.255
**Respondent age (years)**						
< 30	88	65	73.9	Referent	Referent	-
30–39	58	34	58.6	0.49	0.23–1.02	0.056
40–49	49	25	51.0	0.37	0.17–0.81	0.012
50–59	60	37	61.7	0.60	0.29–1.25	0.172
60+	105	50	47.6	0.34	0.18–0.63	0.001
**Diarrhoeal episode in the household in the past two weeks**						
No	296	162	54.7	Referent	Referent	-
Yes	78	54	69.2	1.86	1.09–3.17	0.022
**Basin/tap for hand washing**						
Same room as toilet	193	103	53.4	Referent	Referent	-
Different room to toilet	181	113	62.4	1.47	0.97–2.23	0.066
**International Wealth Index**	-	-	-	1.00	0.98–1.02	0.912

Note: International Wealth Index: Median = 86.3, IQR = 79.1-92.1.

OR, odds ratio; CI, confidence interval; IQR, interquartile range.

Although there was no difference in performance of hand hygiene at critical times between respondents with taps located inside or outside the room housing the toilet, respondents with taps located outside the room housing the toilet were less likely to use soap when washing hands (52.8% vs. 74.6%; OR = 0.38; 95% CI: 0.25–0.59; *p* = 0.000).

## Discussion

Pre-COVID-19 hand hygiene practices in the surveyed population were poor, with only 42% of respondents reporting adequate hand hygiene (always washing their hands with soap and water at critical times).

Inadequate hand hygiene was specifically associated with not using soap for hand washing, although respondents reported good compliance in terms of washing at critical times. Waterless hand sanitiser was not used in this community pre-COVID-19, although the availability and use of such products may have since been introduced as part of pandemic mitigation. It is unlikely that the use of hand sanitiser would be a sustainable practice in low- and middle-income communities outside of pandemic conditions, especially in urban communities with good access to soap and piped water. Households reporting inadequate hand hygiene practices were more likely to have had a diarrhoeal episode in the past two weeks, in keeping with findings from previous studies.^5,6^ This study may have been subject to response bias because of reliance on respondents to answer questions accurately rather than through observation. The importance of drying hands after washing could not be analysed as these data were not collected during the survey. Despite these limitations, we were able to highlight gaps regarding hand hygiene practices in this community.

Younger respondents (< 30 years) were more likely to practice inadequate hand hygiene than older respondents (> 30 years), a finding also made in a study on risk perception and preventive behaviour during the COVID-19 pandemic in South Africa.^11^ All surveyed households had access to piped water on their property, but just under half had a tap located outside the room housing the toilet. Those who did not have a tap in the room housing the toilet were less likely to use soap when washing their hands. Tailored educational messaging should be targeted at young adults in particular, perhaps with programmes introduced at schools and learning centres, particularly through media relevant to a young audience. The importance of using soap for hand hygiene must be emphasised for all age groups. In particular, there is a need for messaging with low-cost, practical solutions (such as soap slings) to encourage keeping soap at taps located outside dwellings and at communal taps.

Studies have shown that direct, targeted communication is required to change risk mitigation behaviours, specifically during outbreaks.^12^ Simple messaging around some of the findings from this study may support the maintenance of good hand hygiene in the long term. A variety of modalities (including peer health educators, multimedia, audiovisual and social media interventions) have been shown to be effective in delivering RCCE during virus outbreaks^12^ and should be utilised for hand hygiene messaging. Effective RCCE for hand hygiene is especially important as COVID-19 pandemic fatigue lowers risk perceptions^2,12^ and should expand to focus on preventing the spread of multiple infectious diseases.
